# Ultra high frequency ultrasound enables real-time visualization of blood supply from chorioallantoic membrane to human autosomal dominant polycystic kidney tissue

**DOI:** 10.1038/s41598-024-60783-3

**Published:** 2024-05-02

**Authors:** Jan Schueler, Jonas Kuenzel, Anna Thuesing, Eric Pion, Rose Yinghan Behncke, Rene Haegerling, Dieter Fuchs, Andre Kraus, Bjoern Buchholz, Boqiang Huang, Dorit Merhof, Jens M. Werner, Katharina M. Schmidt, Christina Hackl, Thiha Aung, Silke Haerteis

**Affiliations:** 1 Institute for Molecular and Cellular Anatomy, University of Regensburg, 93053 Regensburg, Germany; 2 Research Group ‘Lymphovascular Medicine and Translational 3D-Histopathology’, Institute of Medical and Human Genetics, Charité-Universitätsmedizin Berlin, 13353 Berlin, Germany; 3 Berlin Institute of Health at Charité-Universitätsmedizin Berlin, BIH Center for Regenerative Therapies, 13353 Berlin, Germany; 4 Research Group ‘Development and Disease’, Max Planck Institute for Molecular Genetics, 14195 Berlin, Germany; 5 Berlin Institute of Health at Charité-Universitätsmedizin Berlin, BIH Academy, Clinician Scientist Program, 10117 Berlin, Germany; 6 FUJIFILM VisualSonics, Inc., 1114 AB Amsterdam, The Netherlands; 7 Department of Nephrology and Hypertension, Friedrich-Alexander University Erlangen-Nürnberg, 91054 Erlangen, Germany; 8 Institute of Image Analysis and Computer Vision, Faculty of Informatics and Data Science, University of Regensburg, 93053 Regensburg, Germany; 9 Department of Surgery, University Hospital Regensburg, 93053 Regensburg, Germany; 10 Faculty of Applied Healthcare Science, Deggendorf Institute of Technology, 94469 Deggendorf, Germany

**Keywords:** Translational research, Kidney, Polycystic kidney disease

## Abstract

Ultra high frequency (UHF) ultrasound enables the visualization of very small structures that cannot be detected by conventional ultrasound. The utilization of UHF imaging as a new imaging technique for the 3D-in-vivo chorioallantoic membrane (CAM) model can facilitate new insights into tissue perfusion and survival. Therefore, human renal cystic tissue was grafted onto the CAM and examined using UHF ultrasound imaging. Due to the unprecedented resolution of UHF ultrasound, it was possible to visualize microvessels, their development, and the formation of anastomoses. This enabled the observation of anastomoses between human and chicken vessels only 12 h after transplantation. These observations were validated by 3D reconstructions from a light sheet microscopy image stack, indocyanine green angiography, and histological analysis. Contrary to the assumption that the nutrient supply of the human cystic tissue and the gas exchange happens through diffusion from CAM vessels, this study shows that the vasculature of the human cystic tissue is directly connected to the blood vessels of the CAM and perfusion is established within a short period. Therefore, this in-vivo model combined with UHF imaging appears to be the ideal platform for studying the effects of intravenously applied therapeutics to inhibit renal cyst growth.

## Introduction

Autosomal dominant polycystic kidney disease (ADPKD) is the most common monogenetic kidney disease with an incidence of 1:400–1:1000^1,2^. Mutations of the polycystic kidney disease 1 (*PKD1*) or polycystic kidney disease 2 (*PKD2*) gene^3^ lead to the formation of numerous cysts in both kidneys^4^. These cysts progressively inhibit kidney function which results in end-stage renal disease (ESRD) over time^2,5^. Currently, only symptomatic treatment of the disease is possible, such as the reduction of cyst growth, the adjustment of blood pressure, and the treatment of proteinuria^6^. For improved disease management and discovery of new therapeutic targets, a better understanding of the molecular background of ADPKD is necessary. Therefore, a novel 3D-in-vivo model based on the chorioallantoic membrane (CAM) model was established by Bichlmayer et al. that enables the investigation of human ADPKD tissue for several days^7^. In this study, human ADPKD kidney specimens obtained from nephrectomies were grafted onto the CAM and remained vital throughout the experimental period.

The CAM is an extraembryonic membrane within fertilized chicken eggs that is formed between the 3rd and 10th day of embryonic development^8,9^. It functions as the respiratory organ of the embryo and therefore has a physiologically high density of microvessels^9,10^. The development of the CAM vessels progresses in three separate phases. Initially, sprouting of the capillary network occurs between the 5th and 7th day of embryonic development, followed by intussusceptive microvascular growth (IMG) between the 8th and 12th day. Until day 10, the vascular endothelial cells present immature and undifferentiated cells characterized by a high mitotic rate. The resulting capillary network grows through the insertion of transcapillary tissue posts, resulting in the development of new intercapillary tissue profiles that ultimately form full-size intercapillary meshes^11^. Due to its dense network of blood vessels, the CAM is considered a high-level angiogenesis model that provides ideal conditions for the engraftment of tumor tissue or cells^12^. During the cultivation period, multiple therapeutics can be applied to the tissue and different read-outs regarding growth, perfusion, and others can be performed.

One central aspect of this innovative model is the vitality of the cystic tissue which strongly depends on adequate levels of perfusion. Hereby, the exact mechanism of nutrition and gas exchange shortly after engraftment presents a challenging research question. The most common assumption currently is that the tissue receives nutrition via diffusion. On the other hand, perfusion via the CAM vessels to the tissue has also been suggested, but the development of anastomoses or the blood flow itself over a time course of several days has not yet been observed in real-time. Histological staining does not enable any conclusions regarding the level of blood supply or its monitoring over the testing period. Observations on how rapidly the anastomoses between human ADPKD tissue and the CAM vessels are formed may allow to draw further conclusions on the molecular characteristics of this disease and the discovery of new therapeutic targets. Therefore, the objective of this study was to establish UHF ultrasound as a suitable method for monitoring and visualizing renal perfusion in the CAM model to analyze how fast perfusion contributes to the supply of nutrients and oxygen to the human ADKPD tissue.

Several imaging techniques with various advantages and disadvantages can be used to monitor the tissue’ growth, angiogenesis, and perfusion during the study period on the CAM. X-ray methods are perfectly suited for the documentation of vascular characteristics such as blood flow, blood volume, and mean transit as well as microvascular permeability^13^. Besides the ionizing radiation, the injection of contrast agents also poses a risk to the embryo. Another X-ray-based procedure is digital subtraction angiography (DSA). This method, however, can only detect vessels with a diameter greater than 100 µm^14^. In contrast to Computed tomography (CT), magnetic resonance imaging (MRI) does not subject the tissue to ionizing radiation, but also has the drawback of a spatial resolution of 100–500 µm in clinical devices^15^. optical techniques, such as Laser Speckle Contrast Imaging (LSCI) appear as potential alternatives and can be used to investigate angiogenesis and tissue perfusion. Hereby, LSCI identifies moving erythrocytes through the detection of scattered laser light which provides information on blood flow dynamics^7,16^. Also, automated image analysis applications such as the CAM Assay application (KML Vision, Graz, Austria) on the IKOSA platform, can provide additional data regarding angiogenesis and perfusion based on machine learning. The used convolutional neural network (CNN) is specifically designed for the CAM vasculature and calculates different parameters of the blood vessels, such as length, diameter, and branching points^17^. In comparison to the above-mentioned imaging methods, UHF ultrasound (defined as peak frequencies exceeding 40 MHz) as an imaging technique enables a higher resolution of microvessels which is essential for the in ovo visualization of CAM vasculature. There is no depth limitation regarding implanted tissue and the vascularization signal is not affected by depth attenuation. In addition, lower time requirements, lower costs, and real-time imaging, combined with the accessibility of ultrasound devices facilitate regular examinations. Further, the tissue is not exposed to ionizing radiation or potentially damaging contrast agents. The UHF ultrasound method used in this study relied on imaging with a linear array transducer and operated at a peak frequency of 70 MHz. This unique setup allows for real-time imaging at a resolution of 30 µm which cannot be achieved with any other clinically approved ultrasound device to our knowledge. Also, UHF ultrasound used in this study has previously been able to provide highly detailed imaging that was validated by histological analysis^18^.

In addition, tissue perfusion can also be visualized using near-infrared fluorescence imaging after Indocyanine green (ICG) injection into a blood vessel of the CAM. ICG is a cyanine dye that has been used for diagnostic purposes since the 1950s as an indicator substance (e.g. in fluorescence angiography). Due to its chemical structure, ICG fluoresces when stimulated with light in the near-infrared wavelength range (780–3000 nm)^19^. This fluorescence can be visualized with an appropriate near-infrared fluorescence camera. To complement the multimodal imaging approach for studying vasculature and tissue architecture, light sheet microscopy was applied on tissue samples to perform whole-mount 3D histology. In contrast to physical sectioning, this optical approach allows the generation of a series of optical sections from immunofluorescence-stained, optically cleared tissue samples. By subsequent digital 3D reconstruction of the image data, the entire vasculature is visualized in its spatial context. Thus, light sheet imaging-based 3D histology represents a tool for vascular phenotyping and in-depth investigation of vascular anastomoses between human and avian blood vessels.

An additional aspect of the CAM model as an in vivo model is the incorporation of the 3R principle (“replace, reduce, refine”) for the prevention of unnecessary suffering of animals used for research purposes. In addition to its simple handling, low cost, and versatility, the CAM model also bridges the gap between preclinical and clinical studies. In combination with UHF imaging, the CAM model is expanded significantly and improved as an angiogenesis model to gain further insights into the molecular mechanisms of ADPKD and potentially many more diseases.

## Methods

### Preparation of cystic tissue

Human renal cystic tissue was obtained from ADPKD patients who received an elective nephrectomy at the Department of Surgery, University Hospital of Regensburg. Written consent was obtained from all patients and experiments were approved by the ethics committee of the University of Regensburg (no. 20-1886-101). After the dissection of tissue containing microcysts, these were weighed and subsequently grafted onto the CAM. Tissue samples were placed in the center of agarose rings to position them in the center on the CAM.

### 3D-in-vivo-model—the CAM model

The CAM model was performed as described previously^7,17,20^. In short, after an incubation period in the ProCon incubator (Grumbach, Asslar, Germany) of 4 days, a window was cut into the shell of fertilized chicken eggs and sealed temporarily with Leukosilk® (BSN medical GmbH, Hamburg, Germany). Human renal cystic tissue with microcysts was grafted onto the CAM between the 7^th^ and 10^th^ day of embryonic development. Daily macroscopic images of the engrafted tissue were taken (Leica® M205A microscope), and eggs were checked for vitality. After the study period (12 h or 7 days), the tissue was removed and stored in 4% PFA in PBS for 24 h.

### Ultra high frequency ultrasound measurements

To monitor perfusion and vasculature of the renal cystic tissue, UHF ultrasound images were obtained at regular intervals. 14 engrafted CAMs were examined daily, 14 every other day. For this purpose, the Vevo® MD UHF ultrasound system (FUJIFILM VisualSonics Inc., Toronto, Canada) was used with the UHF70 ultrasound transducer at a frequency of 71 MHz. The Vevo MD is the first and only UHF clinical ultrasound system that provides an unprecedented resolution of 30 µm at a maximum penetration depth of 10 mm. This enables visualization of microvessels at high resolution in B-Mode without using a Power Doppler, advanced imaging or analysis protocols, or the use of contrast agents. For optimal UHF image quality, 2 ml of 0.9% NaCl was temporarily distributed on the CAM and removed after the image acquisition was completed. Videoclips with a length of 6 s were acquired for further analysis.

### Semi-automatic blood vessel annotation

**Figure 1:**
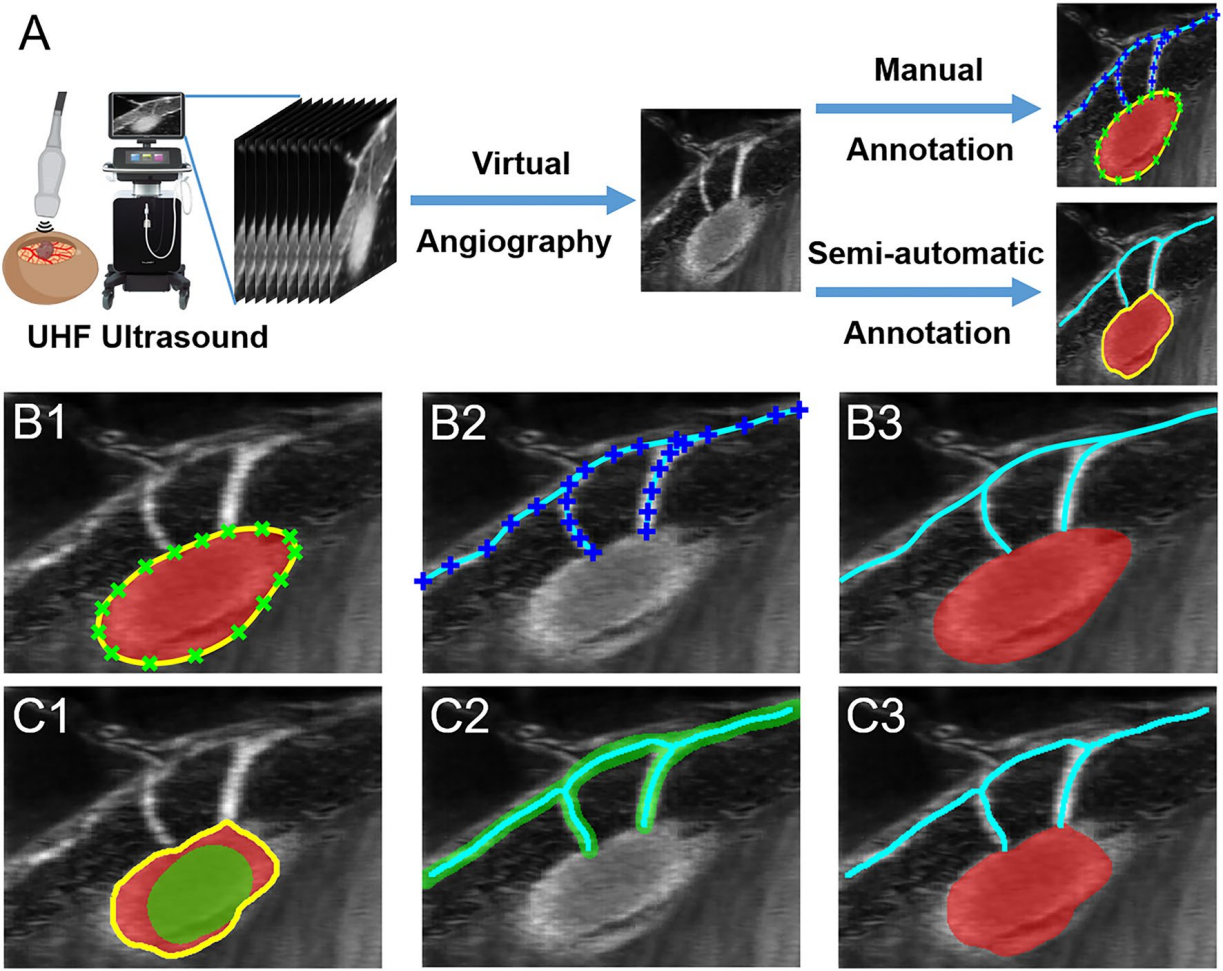
An illustration of a self-developed interactive auxiliary app. (**A**) A virtual angiography is applied to the video recorded by the Vevo® MD UHF ultrasound system to obtain a vessel-augmented image for manual annotation and semi-automatic annotation. (**B1**–**B3**) With manual annotation, the smooth boundary (in yellow) of the CAM vessel and the microvessel curves can be obtained by interpolating corresponding annotation points (in green x and blue +, respectively). (**C1**–**C3**) In semi-automatic annotation, the smooth boundary (in yellow) of the CAM vessel (in red) is obtained by applying an active contour model (or snake algorithm) with a manually initialized annotation mask (in green). The microvessels (in cyan) are obtained by calculating the skeleton of manually initialized annotation mask (in green).

To reduce the speckle noise while highlighting both microvessels and CAM vessels from the surrounding background, a digital subtraction angiography was applied to the recorded videoclips. Afterwards, either manual or semi-automatic annotations were implemented (Fig. 1). In the manual annotation, both CAM vessel boundaries and microvessel curves were interpolated based on annotated points (Fig. 1 B1–B3). Regarding the semi-automatic annotations, CAM vessels were obtained by using an active contour model with roughly annotated region-of-interest (ROI) areas (Fig. 1 C1–C3). The microvessel curves were obtained by detecting the skeleton of manually annotated vessel areas^21^.

Suppose that the recorded ultrasound video is a temporal image set ${\mathbf{I}}_{{{\text{US}}}} : = \left\{ {I_{1} ,I_{2} , \ldots ,I_{T} } \right\}, \;\;I_{t} \in {\mathbb{R}}^{M \times N} ,\;\; t = 1,2, \ldots ,T$, the absolute finite difference of ${\mathbf{I}}_{{{\text{US}}}}$ is defined as $\left| {\vartriangle {\mathbf{I}}_{{{\text{US}}}} } \right|: = \left\{ {\left| {\vartriangle I_{1} } \right|,\left| {\vartriangle I_{2} } \right|, \ldots ,\left| {\vartriangle I_{T - 1} } \right|} \right\}$, where $\vartriangle I_{t} : = I_{t + 1} - I_{t}$. Ideally, one could assume that the background of successive ultrasound image frames to be identical. Therefore, the absolute finite difference $\left| {\vartriangle I_{t} } \right|$ implies the reflection difference to the ultrasound mainly due to the blood flow in both microvessels and CAM vessels. Accumulating these differences highlighted the location of blood vessels while reducing possible interferences from the background.

The desired virtual angiography of ${\mathbf{I}}_{{{\text{US}}}}$, $I_{{{\mathbf{VA}}}} \in {\mathbb{R}}^{M \times N}$, can be simply obtained by $I_{{{\mathbf{VA}}}} : = \mathop \sum \limits_{t = 1}^{T - 1} \left| {\vartriangle I_{t} } \right|/\left( {T - 1} \right)$ or an online update formula $I_{{{\mathbf{VA}},t}} : = \left( {\left( {t - 1} \right) I_{{{\mathbf{VA}},t - 1}} + \left| {\vartriangle I_{t} } \right|} \right)/t$, where $I_{{{\mathbf{VA}},t - 1}}$ and $I_{{{\mathbf{VA}},t}}$ represent the instant virtual angiography at time $t - 1$ and $t$.

### Indocyanine green fluorescence

ICG-monosodium salt (VERDYE®) was solved in purified water (0.05 mg/ml). Then, 0.05 ml of this solution was injected into a prominent vessel of the CAM. The Elevision IR (VSIII) fluorescence system (Medtronic®, Germany) was used for fluorescence imaging in perfusion recognition modes to visualize and trace the injected ICG. A sterile 1 ml single-use syringe (Henke-Ject®, Henke-Sass, Wolf GmbH, Tuttlingen, Germany) with a sterile 33G 0.20 × 4 mm hypodermic needle (Mesoram®, RI.MOS SRL, Mirandola, Italy) was used for injection.

### Histochemistry

The removed tissue samples were stored in 4% PFA in PBS for 24 h for fixation and subsequently stored in a solution with 0.02% sodium-azide. After dehydration in an automated process for 26 h and paraffin embedding, the tissue was cut into 6 µm-thick sections. All samples were first deparaffinized and hydrated in a graded alcohol series prior to staining with H&E according to the standard protocol. For detection of human and chicken CD31, sections were deparaffinized and boiled for 3 min in Target Retrival Solution (S1699, Agilent, Waldbronn, Germany) followed by incubation with avidin and biotin for 20 min, respectively. After incubation in 3% hydrogen peroxide solution for 15 min and application of blocking buffer, the sections were incubated overnight with the primary antibodies mouse α CD31 (GA619, Dako Omnis, Hamburg, Germany; 1:500) or rat α CD31 (DIA-310, Dianova, Hamburg, Germany; 1:500). Signals were visualized using an ABC kit (Vector PK6100), TSA kit (AKOYA nEL749A001KT) followed by streptavidin horseradish peroxidase amplification (abcam ab64269) according to the manufacturer's instructions. For VEGF-A staining, the same protocol as described for CD31 was applied using the primary mouse α VEGF-A antibody (ab1316, Abcam, Cambridge, UK).

### Whole-Mount immunofluorescence stainings

Avian blood vessels of the CAM were injected with Isolectin GS-IB4 conjugated with Alexa FluorTM 647 (I32450, Invitrogen®, Thermo Fisher Scientific, Waltham, MA, United States) to label all endothelial cells connected to the avian blood circulation. PFA-fixed tissue samples were washed twice with PBS followed by permeabilization (0.5% TritonTM X-100 in PBS) and blocking with PermBlock solution (1% BSA, 0.5% Tween® 20 in PBS) at 4 °C. Subsequently, tissue samples were wholemount immunofluorescence stained using CD31 Antibodies (unpublished) directly coupled with Alexa Fluor 488 and mouse monoclonal IgG1a anti-SMA antibody directly labeled with Cy3 (C6198, Sigma-Aldrich®, Merck, Darmstadt, Germany) in PermBlock. Following the staining process, samples were washed three times with PBS-T (0.1% Tween® 20 in PBS) and dehydrated in increasing methanol concentrations (50%, 70%, 95%, > 99.0%, and > 99.0% methanol in ddH2O). Finally, samples were optically cleared twice in a benzyl alcohol/benzyl benzoate solution (BABB, ration 1:2) before being studied on a Zeiss Lightsheet 7 microscope (Zeiss, Oberkochen, Germany) at various magnifications and an optimal step size depending on the magnification (ranging from 1.8 to 5.95 mm). The acquired light sheet image stacks were reconstructed in 3D using the Microscopy Image Analysis Software Imaris (Oxford Instruments, Abingdon, United Kingdom).

### Ethics approval and consent to participate

Informed consent was obtained from all subjects and/or their legal guardians and experiments were approved by the ethics committee of the University of Regensburg (no. 20-1886-101). All patient data gets encrypted, and every patient gets an internal patient ID. All data protection guidelines from the University of Regensburg are met.

## Results

### UHF ultrasound imaging shows blood flow through an anastomosis from chicken to human cystic kidney tissue 12 h after engraftment

**Figure 2:**
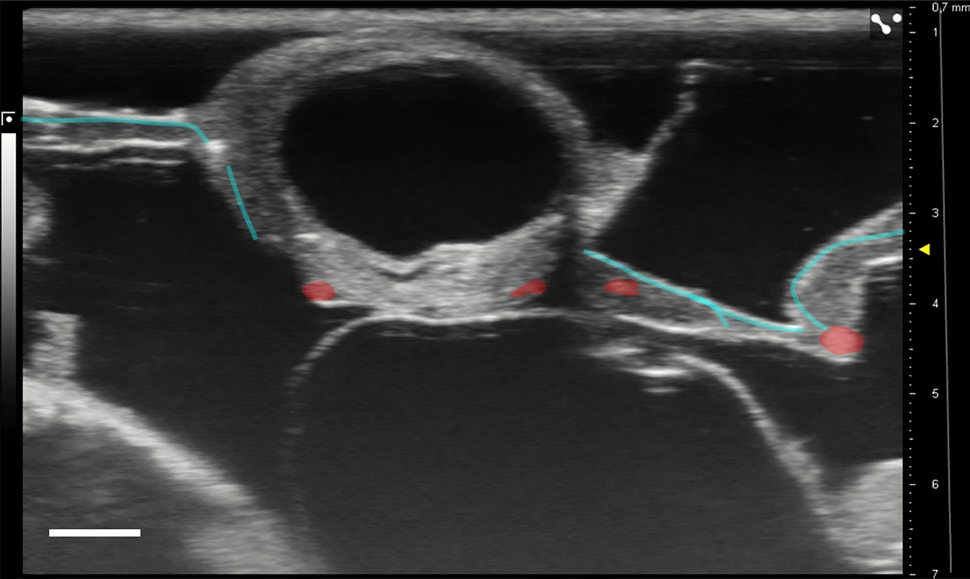
UHF ultrasound image of microcystic tissue 12 h after engraftment. Microcysts with microvessels (cyan) penetrating from the right and left into the lower part of the engrafted tissue measured with UHF ultrasound imaging. Larger vessels of the CAM are highlighted in red (Supplementary Video 1). Scale bar represents 1 mm. The gray scale (left) shows the available gray tones. The millimeter scale (right) shows the penetration depth. The gray line (right) shows the time gain compensation (TGC): a way to overcome ultrasound attenuation is time gain compensation, in which signal gain is increased as time passes from the emitted wave pulse. This correction makes equally echogenic tissues look the same even if they are located in different depths.

**Figure 3:**
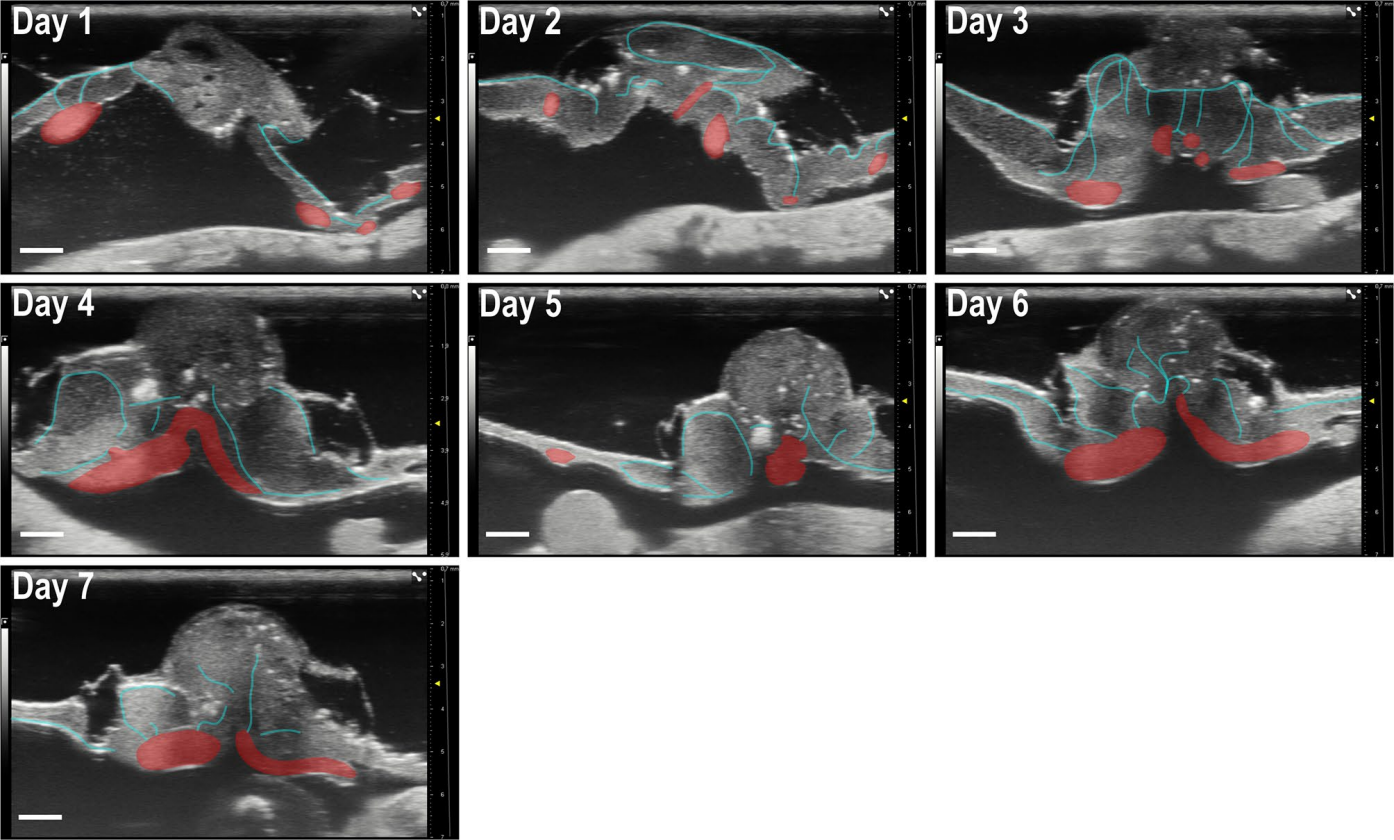
UHF ultrasound images taken daily during the incubation period of seven days. Daily images from day 1 to day 7 show an increasing number of microvessels (cyan) inside the cystic tissue reflecting the process of angiogenesis. Larger vessels of the CAM are highlighted in red. (Supplementary Video 2–8). Scale bar represents 1 mm.

UHF ultrasound was used to visualize microcysts dissected from a human ADPKD kidney 12 h (Fig. 2) after grafting onto the CAM. The ultrasound videos of the blood flow were highlighted via virtual angiography and can be found in the Additional files (see Supplementary Video 1). In the beginning, larger blood vessels of the CAM were seen underneath the tissue from which smaller microvessels grow into the cystic tissue. The CAM and connected vessels were identified by UHF which visualized the movement of speckles, based on the motion of the nucleated avian erythrocytes (Fig. 3 Day 1–Day 7). Imaging focused on the center of the implanted tissue which enabled a real-time and non-invasive assessment of an increase in vascularization and change of cyst size. Over the 7-day experimental period, an increasing number of branching points were formed between human tissue and CAM blood vessels (see Supplementary Video 2–8). Smaller microvessels branching from larger blood vessels were identified that penetrated into the cystic tissue (Fig. 3 Day 1–Day 7).

### Invasion of chicken erythrocytes into human cystic tissue 12 h after grafting

**Figure 4:**
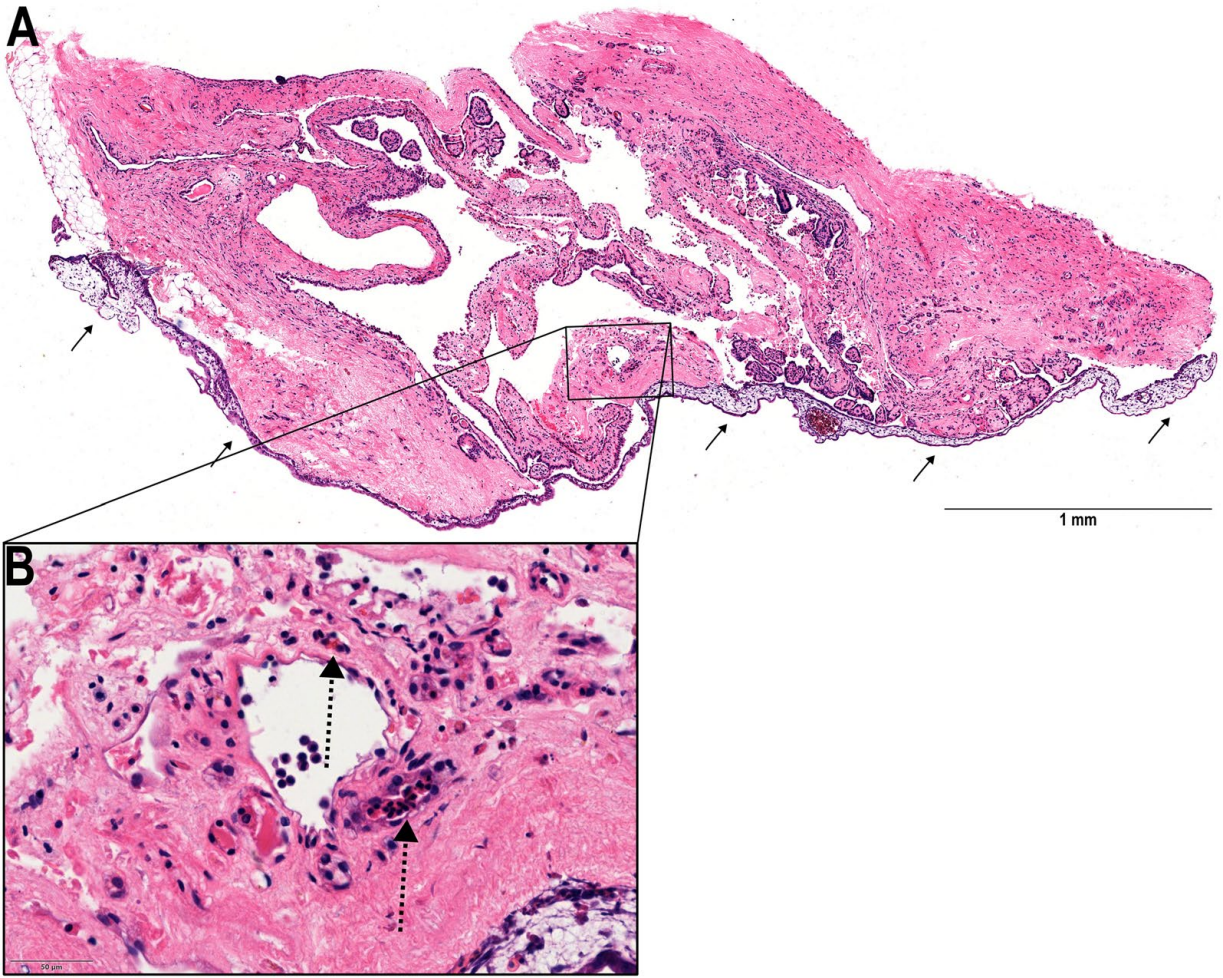
H&E stained histological section 12 h after engraftment. (**A**) HE stained section shows functional attachment to the CAM (black arrows) and therefore appears vital with no significant signs of necrosis. (**B**) Magnification of the area marked in (**A**) showing a microcyst with nucleated erythrocytes of the chick embryo within vessels (dashed arrows).

**Figure 5:**
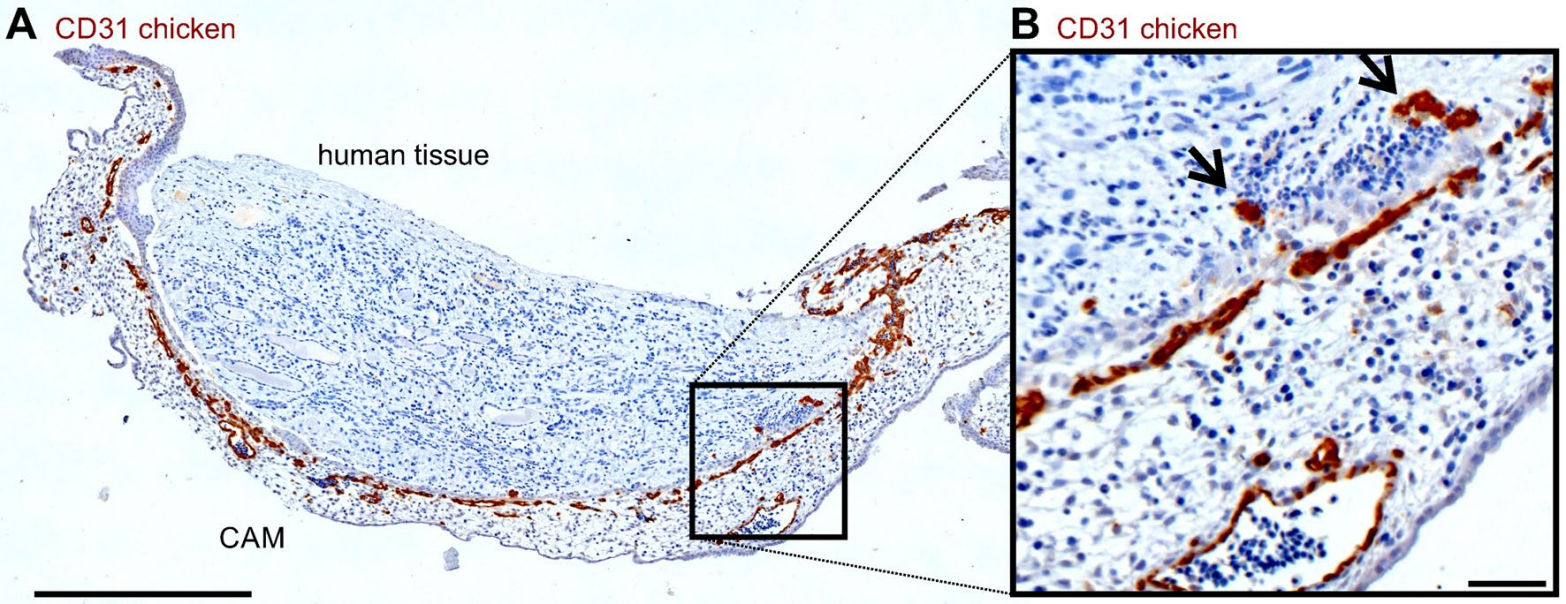
CD31 staining of a CAM sample 12 h after grafting for differentiation of human and chicken vessels within the tissues. (**A**) Human cystic tissue and CAM were stained for chicken CD31. Scale bar represents 500 µm. (**B**) Magnification of the area marked in (**A**) indicates positive chicken CD31 signals within the human tissue (arrows). Scale bar represents 50 µm.

**Figure 6:**
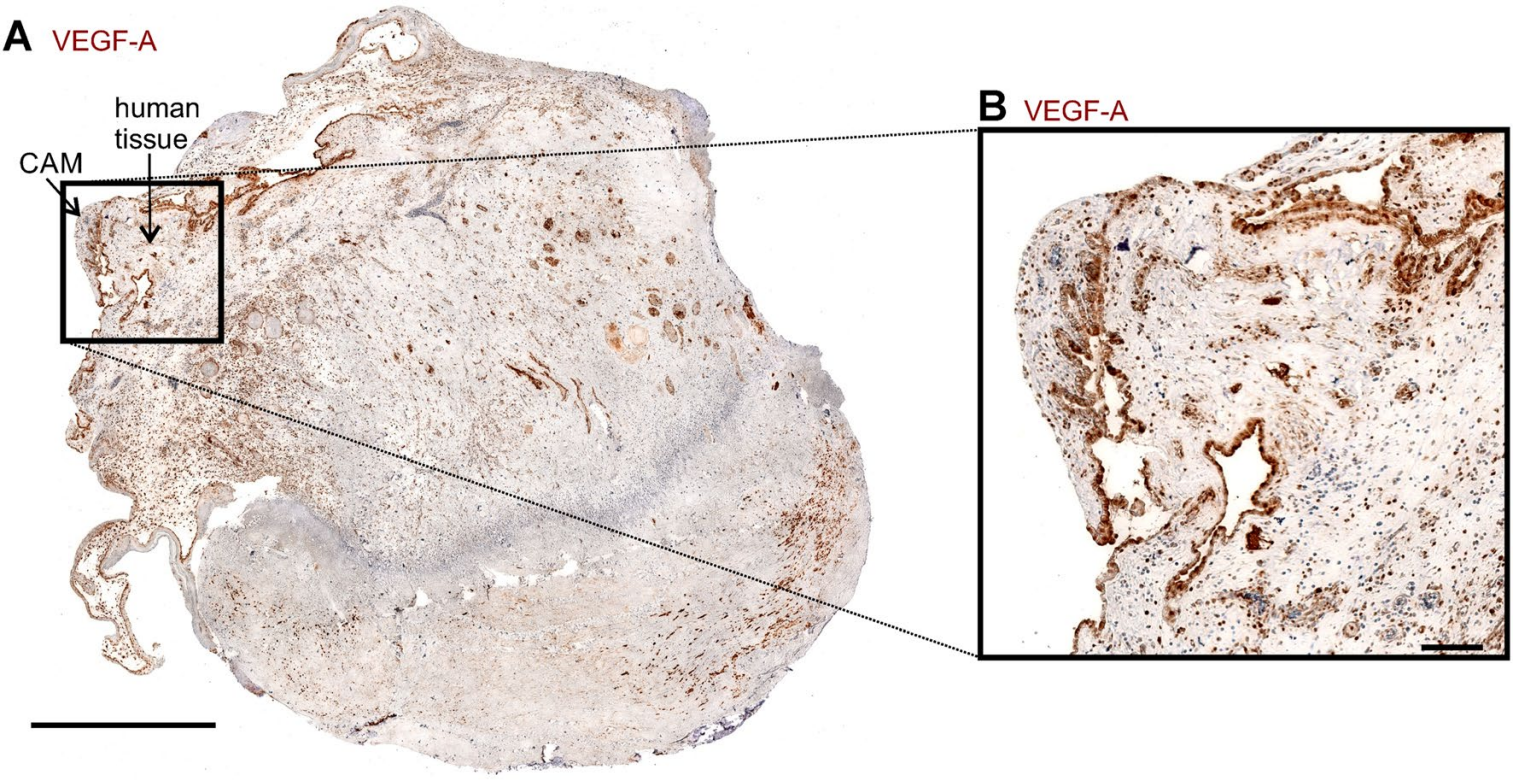
VEGF-A staining of a CAM sample 12 h after grafting. (**A**) CAM tissue was stained for VEGF-A 12 h after engraftment, indicating VEGF-A expression within the CAM (arrow) and human tissue, respectively. Scale bar represents 1 mm (**B**) Magnification of the area marked in (**A**). Scale bar represents 100 µm.

Cystic tissue (Fig. 2) was removed from the CAM for immunohistochemical analysis 12 h after grafting (Fig. 4A). Blood supply from the CAM was confirmed by H&E-stained histological sections revealing nucleated erythrocytes from the chicken embryo within the human renal cystic tissue (Fig. 4B). In line with these findings, chicken-derived vessels were found in the human tissue using anti-chicken CD31 antibody (Fig. 5A, B). In addition, the vascular endothelial growth factor A (VEGF-A) was present in both, human tissue, and the CAM (Fig. 6A, B). The sections showed a functional anastomosis of the engrafted human tissue and the CAM, whereby the graft is supplied through the chicken embryo’s blood vessels.

### Anastomoses between chicken and human blood vessels in human cystic kidney tissue 12 h post-grafting

**Figure 7:**
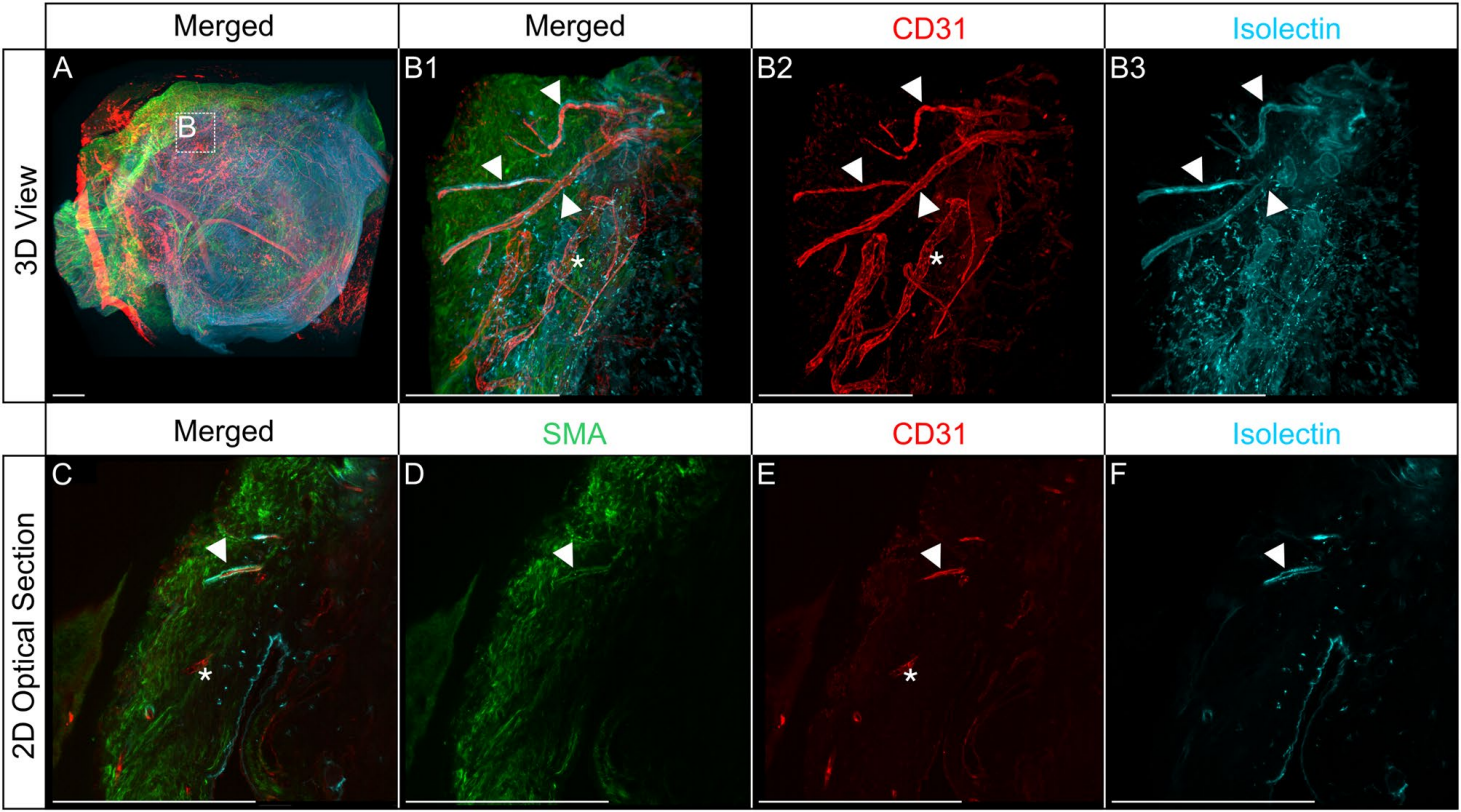
Immunofluorescence staining demonstrated the presence of anastomoses between chicken and human blood vessels in human cystic kidney tissue 12 h post-grafting. Wholemount preparations of human cystic kidney tissue were stained for human CD31 (red), SMA (green), and Isolectin GS-IB4 (cyan). (**A**, **B**) 3D reconstructions. Selected magnifications (framed area) highlight regions with human CD31 positive vessels (**B2**) and Isolectin GS-IB4 (**B3**) positive vessels; a merged view of the single channel is shown in (**B1**). (**C**–**F**) 2D optical sections of the 3D reconstructions. Double-positive blood vessels with human CD31 and Isolectin GS-IB4 are indicated by arrowheads. Asterisks demonstrate blood vessels that were only positive for staining with human CD31. Scale bars represent 500 µm.

**Figure 8:**
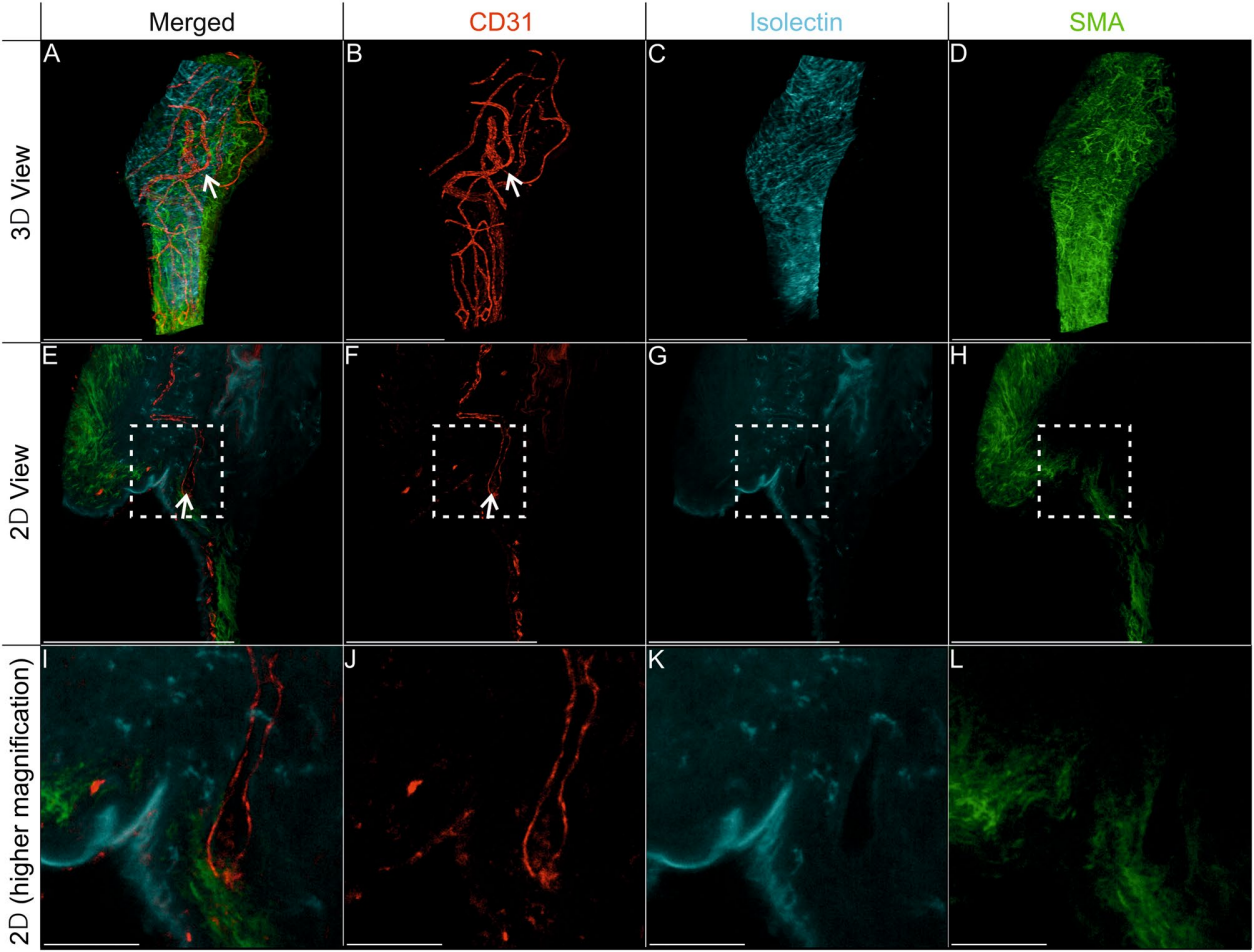
Invasion of chicken erythrocytes into human CD31-positive blood vessels in human cystic tissue 12 h after grafting. Human cystic kidney tissue wholemount preparations stained for human CD31 (red), SMA (green), and isolectin GS-IB4 (cyan). (**A**–**D**) 3D reconstructions. (**E**–**H**) 2D optical sections from the 3D reconstructions. Selected magnifications (framed area) highlight regions with chicken erythrocytes in human CD31-positive blood vessels (**I**–**L**). Human CD31-positive blood vessels containing chicken erythrocytes are indicated by arrowheads. (**A**–**H**) Scale bars correspond to 500 µm. (**I**–**L**) Scale bars correspond to 75 µm.

To further investigate the vascular connection site between avian and human blood vessels in the CAM model, whole-mount immunofluorescence staining of the chimeric tissue samples was performed after injecting fluorescence-labeled Isolectin GS-IB4 into the embryonic blood circulation. Stained tissue was then optically sectioned using a light sheet microscope to visualize the vasculature in its entirety (Figs. 7 and 8). Using this approach, endothelial cells of avian origin were marked by anti-chicken CD31 antibody as well as Isolectin GS-B4, which was injected into the blood circulation of the egg and stained all blood vessels connected to the avian blood circulation. Human endothelium was marked with anti-human CD31 (hCD31) nanobody, which does not show cross-reactivity with chicken CD31 (Figs. 7A, B1, C, 8A, E, I). This enabled the determination of areas of vascular anastomosis between both vascular systems by hCD31 nanobody staining and Isolectin B4 signal (Fig. 7B2, B3, E, F). For better visualization of the non-positive human CD31 vessels, they were identified with smooth muscle antibody (SMA) (Fig. 7D, 8D, H, L). In addition, nucleated red blood cells derived from the chicken embryo were detected within hCD31-positive, Isolectin GS-IB4-negative blood vessels (Fig. 8A–C, E–G, I–K).

### ICG fluorescence demonstrates blood flow from chicken to human cystic kidney tissue 12 h after engraftment

**Figure 9:**
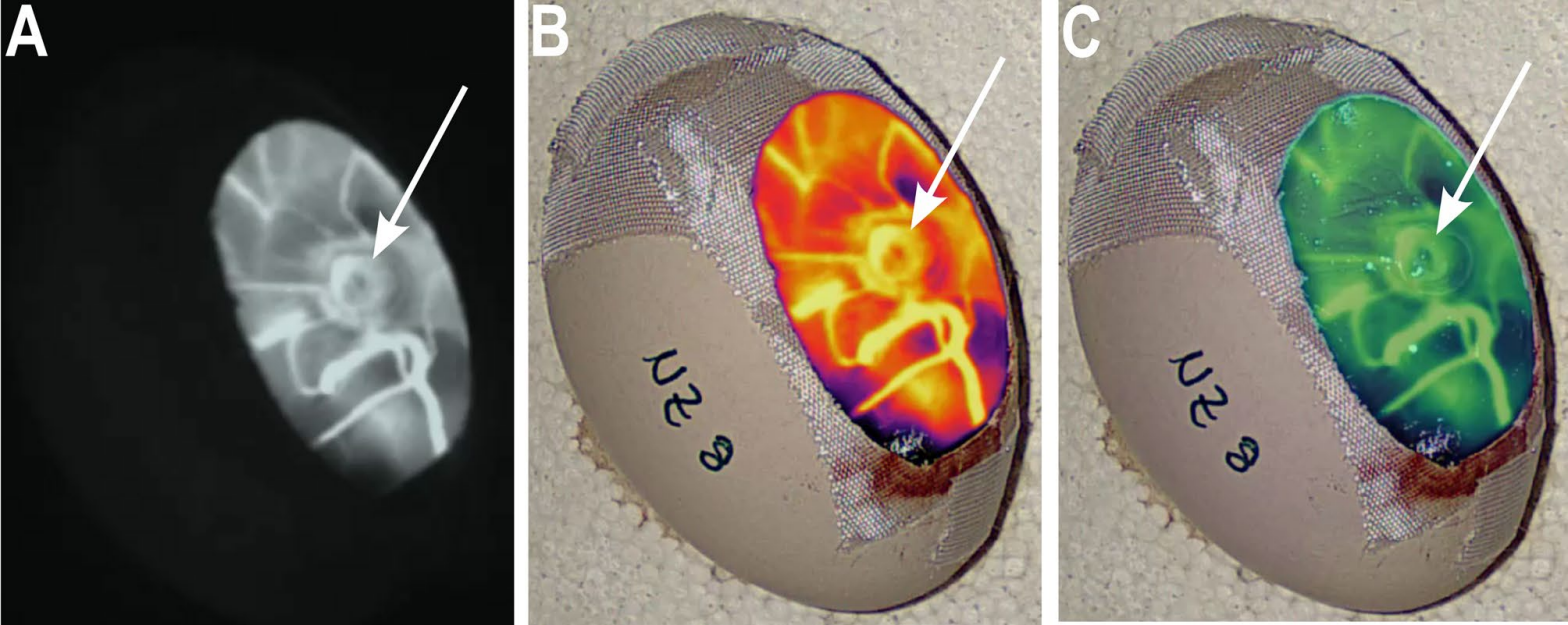
Near-infrared Images after ICG injection into the CAM vessels. In ovo perfusion within the cystic tissue (white arrow). (**A**) Near-infrared image, (**B**) infrared overlay, and (**C**) fluorescent overlay of the CAM model.

12 h after grafting, ICG was injected into a CAM vessel, and its distribution was visualized with an infrared camera (Fig. 9). ICG dispersed within the CAM vessels and subsequently in the human cystic tissue which was shown by fluorescence in-ovo (Fig. 9A–C). According to the blood flow detected by ultrasound and the avian erythrocytes observed in H&E sections, the ICG detection in the human cystic tissue 12 h after engraftment indicates vessel sprouting from CAM to the grafted tissue and the generation of vessel anastomosis in a very short period.

## Discussion

**Figure 10:**
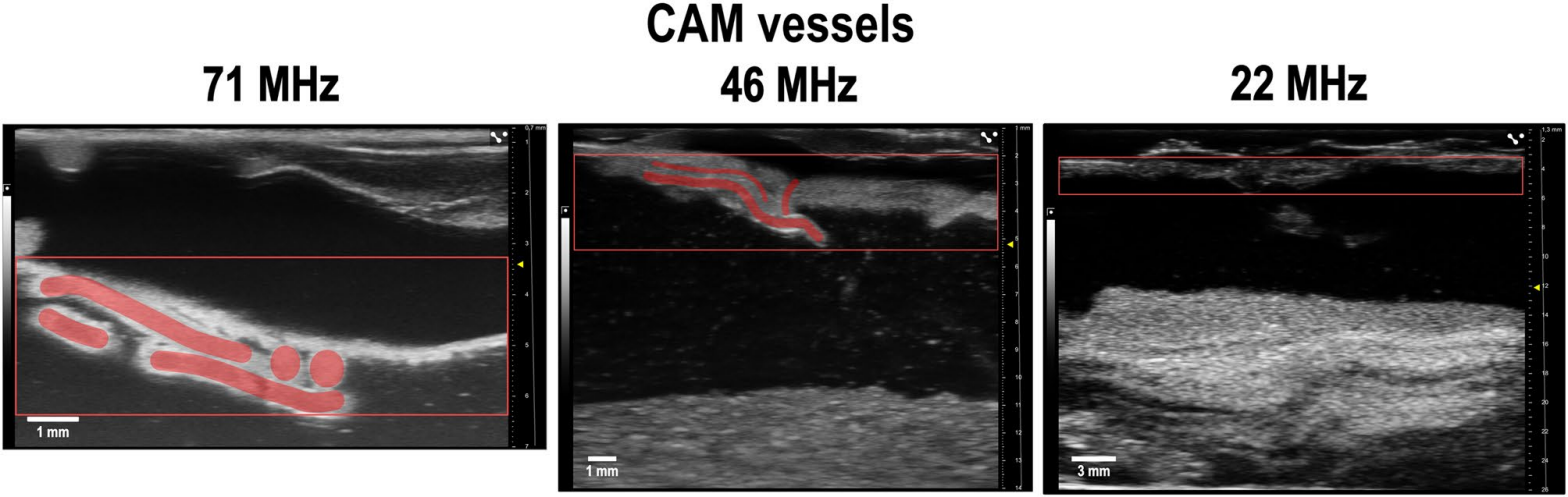
Transducer comparison for imaging of the CAM vasculature. Ultrasound images of CAM vessels were acquired with three ultrasound transducers of different frequency ranges (Supplementary Videos 9–11): UHF70 transducer at a frequency of 71 MHz (central frequency 50 MHz), UHF48 transducer at a frequency of 46 MHz (central frequency 30 MHz) and UHF22 transducer at a frequency of 22 MHz (central frequency 15 MHz). The region of interest is marked with a red box and CAM vessels are highlighted in red.

While ultrasound has been utilized in CAM models, the variability of frequencies makes a direct comparison between to UHF ultrasound close to impossible. While conventional clinical ultrasound uses frequencies around 15 MHz offering deep tissue penetration up to 60 mm, the resulting axial resolution of 0.15 mm is inferior to imaging at 71 MHz (0.030 mm resolution). Figure 10 illustrates the difference in image quality between UHF ultrasound and low frequency ultrasound (LFU) (Supplementary Videos 9–11). LFU Images do not enable a sufficient visualization of the CAM vessels. As such, this work offers a completely novel methodology to monitor human tissue on a CAM using clinically approved equipment. In 2020, Eckrich et al., investigated the growth of liver cancer HuH7 tumor cells on the CAM to evaluate whether ultrasound is suitable for quantifying tumor size, growth, and angiogenesis in the CAM model. In this study, ultrasound images were repetitively acquired in color-duplex mode using the GE Healthcare Ultrasound LOGIQ E9 system (GE Healthcare Little Chalfont, UK) and a 15 MHz linear transducer^22^. In contrast, images acquired at 71 MHz enable the visualization of microvessels and blood flow even without the use of a Power Doppler Mode (PDM) which eliminates several drawbacks: with PDM it is not feasible to differentiate small, functional blood vessels based on moving blood flow without artifacts resulting from the embryo´s movement in the background. Therefore, PDM could not sensitively detect blood flow in real-time to the same extent as UHF in B-Mode could. The clinically approved UHF ultrasound used in this study combines high-resolution anatomical and functional visualization in real-time and is therefore superior to advanced Doppler imaging protocols which are dependent on extensive post-processing of the acquired data^23^.

Huang et al. examined Renca CRL-2947 cell line tumors using ultrasound localization microscopy (ULM) to visualize microvessels more efficiently and with higher resolution. Therefore, images were acquired using a Verasonics Vantage ultrasound system at a frequency of 25 MHz^24^. However, this method is based on the injection of microbubbles and this invasive procedure poses a risk to the embryo. Even if this enables a more precise visualization of microvessels, this method is not suitable for daily screenings due to the time and effort involved.

Power et al., on the other hand, used 3D ultrasound to monitor the effects of chemotherapeutics on H3.1K27M cell line tumors. Here, the investigation was performed using a Vevo 3100 UHF imaging system (FUJIFILM VisualSonics Inc., Toronto, Canada) and a 50 MHz transducer. Images acquired in RF PDM were subsequently analyzed with Vevo LAB software for the quantification of volume and vasculature^23^. While UHF is clearly superior in the visualization of individual microvessels compared to the visualization of blood flow using PDM, a 3D ultrasound enables an exact calculation of tissue volumes that cannot be achieved with UHF ultrasound. Recently, Chu et al., also used 3D ultrasound with 3D PDM to investigate tumor fragments from advanced late-stage ovarian cancer patients and acquired images with the Vevo 2100 (FUJIFILM VisualSonics Inc., Toronto, Canada). Here, cling wrap with transducer gel was placed on the tumor for sound transmission^25^. In this study, the method was refined using 0.9% NaCl without cling wrap to avoid Doppler artifacts which reduce the image quality. In addition, NaCl is easier to handle because it can be kept sterile prior to use, it is easily applied, and removed with a microliter pipette. Furthermore, it is also distributed more evenly on the CAM, which avoids local stress due to the additional weight on it. Another study by Bataghva et al., showed a scalable and efficient method for the modeling of a tumor and evaluation of its treatment response based on perfusion parameters derived from ultrasound image analysis. To demonstrate this concept, 2D- as well as 3D-ultrasound and contrast-enhanced ultrasound (CEUS) were performed on Caki-1 kidney tumor cells, which were grafted onto ex ovo CAMs. 3D Power Doppler images and 2D B-Mode image sequences were acquired using the VisualSonics Vevo 2100 ultrasound system (FUJIFILM VisualSonics Inc., Toronto, Canada) with a 40 MHz linear array, while CEUS imaging was performed using a 20 MHz linear array^26^. Both the injection and the contrast agent itself pose a risk to the embryo which can be eliminated by using UHF imaging.

Recently, Power et al.^23^ reported an increased risk of embryonic lethality using PDM at high frequencies. However, in this study no such adverse events were observed which is in line with previous findings by Eckrich et al.^22^ and Hegemann et al.^27^. This difference could be due to the higher acoustic power used in Doppler imaging at high frequencies. Factors such as infections, temperature fluctuations, or agitation are more likely to cause increased mortality of the embryos than the ultrasound waves themselves. In 2023, Hegemann et al. ^27^ showed that UHF ultrasound can be used as a safe method in cardiovascular studies in the CAM model and complies with the 3R principles, further indicating that UHF ultrasound is safe and causes no harm to the embryos. Because of the low mechanical and thermal indices of the Vevo MD UHF ultrasound imaging system used in our study, an adverse effect of the ultrasound on the tissue can be excluded. The UHF 71 MHz ultrasound probe is clinically used, e.g., in dermatology with no adverse effects reported here as well. Taken together, the described method of assessing the CAM´s blood vessel network and engrafted tissue with clinically approved UHF ultrasound in B-Mode represents a safe, high-resolution, and real-time method that can be widely used without complicated image acquisition or analysis.

While MRI forms an essential tool in clinical oncology, its spatial resolution does not allow to visualize the microvasculature of the CAM. In addition, the acquisition time of MRI is not suitable to assess the vascular properties to the same extent UHF ultrasound offers^28^. CT offers supreme spatial resolution of dense tumor structures such as calcification. However, without adding contrast agents, CT has a limited ability to visualize soft tissue structures making it challenging to assess microvascular networks in the CAM. In addition to the real-time, high-resolution, non-ionizing imaging, UHF ultrasound operates at no running costs which is a key advantage to both CT and MRI imaging. Further, the availability of the UHF ultrasound device allows for flexible experimental planning and fast, reliable, and reproducible daily examinations of the tissue. This enables an ideal protocol for the monitoring of engrafted tissue on the CAM. Angiogenesis, perfusion as well as newly formed microvessels can be visualized and evaluated with this imaging technique throughout the entire experimental period without any manipulation of the CAM. Clinically, dyes such as ICG can be used for visualizing vessels with a near-infrared camera. Even though the use of ICG is clinically approved and visualization in cystic tissue is possible in the described model, this method does not allow optimal visualization of blood vessels in depth or longitudinal imaging in ovo. Furthermore, the risk of injury to the vessels cannot be completely ruled out. It requires experienced technicians to inject the dye precisely and to minimize the risk of bleeding, which would cause severe damage or death to the embryo and could result in exclusion from the experiment.

To further validate the presented results, optical sectioning of the entire CAM was performed using wholemount immunofluorescence staining and light sheet microscopy. In contrast to classical 2D histology, which only enables the analysis of representative 2D sections, and which is always associated with very limited spatial information and the risk of missing anastomoses between human and avian blood vessels, the light sheet imaging-based 3D-histology approach enables analysis of all blood vessel in the sample. Therefore, all anastomoses between chicken and human blood vessels as well as erythrocytes within blood vessels can be identified. CAM vessels were injected with Isolectin B4 and wholemount staining for human endothelium (hCD31) and smooth muscle cells (SMA) was performed to identify anastomoses between both vascular systems. After optical sectioning and analysis of 3D reconstruction as well as all-optical 2D sections, hCD31, and Isolectin B4 double-positive blood vessels were detected. This indicates an anastomosis between human and avian blood vasculature as a non-connected vasculature does not result in double positive stained vessels. To complement this finding the presence of nucleated chicken erythrocytes in human blood vessels was analyzed. As nucleated erythrocytes in hCD31-positive blood vessels were detected at various sites, a general anastomosis mechanism between chicken and human blood vessels supplying the cyst with oxygen and nutrients is very likely. 3D histological analysis of CAMs confirmed the results of classical histology and UHF ultrasound analysis.

However, the formation of anastomoses after transplantation of human tissue samples in in-vivo models has already been observed previously by Kunzi-Rapp et al.^29^ who used the CAM assay to develop a short-term in-vivo system for human skin. In this study, human skin specimens formed anastomoses after 2–3 days and were thus connected to the circulating system of the embryo. Montecinos et al.^30^, on the other hand, investigated the process of angiogenesis of prostate cancer tissue in immuno-compromised mice. Here, the formation of anastomoses only occurred on day 4 after transplantation. The present study found that polycystic kidney tissue developed functional anastomoses to the blood vessels of the chicken embryo within the first 24 h after surgical removal. Whether this rapid formation of anastomoses is associated with characteristics of ADPKD will be investigated in further projects but is beyond the scope of the present study. One target could be the expression of vascular endothelial growth factor-A (VEGF-A), which was detected in cystic endothelial cells in ADPKD patients^31^. VEGF-A affects blood vessels, with VEGF-A_165_ as the main proangiogenic form which supports vascular survival, migration, proliferation, permeability, and dilatation. Furthermore, the growth factor angiopoietin 1 (Ang1) could also lead to endothelial survival and stabilization by binding to the receptor Tie2^32^. Manipulating growth factors and interrupting the pathways triggered by them could be an important target for new therapeutic approaches. However, this approach is still in its early stages and more research is required in this field.

Until now, disease management has primarily focused on improving clinical outcomes and delaying the onset of ESRD^6^. However, UHF imaging in the CAM model resembles a model that is perfectly suited for high-throughput testing of new personalized therapeutic approaches. Currently, human kidney organoids are the most advanced in vitro models that enable the study of disease mechanisms, drug screening, and preclinical testing. However, these models lack important kidney characteristics such as the microenvironment, blood flow, and vascularization. Mouse and rat models, on the other hand, resemble a better physiology that is similar to the human kidney. However, a variety of promising therapies tested in rodent models could not be translated to humans successfully in the past which leads to the conclusion that this approach is not suitable for drug screening regarding ADPKD^33^. The CAM model, on the other hand, combines the possibilities of research into disease mechanisms, drug screening, and preclinical testing, which is more cost-efficient and easier to handle than rodent models, while contributing to the implementation of the 3R principles.

Future projects will focus on the development of parameters that enable the quantification of angiogenesis and perfusion. One possible option would be the development of automated image analysis approaches for the B-scans. AI-supported algorithms could be superior at identifying functional blood vessels and detecting blood flow selectively even in the case of stronger background movement. However, such approaches require large amounts of datasets and annotations to perform well, which would be beyond the scope of this work.

## Conclusion

In this study, clinically approved UHF imaging was established as a new imaging technique in the CAM model and the rapid formation of anastomoses between human polycystic tissue and chicken vessels was observed non-invasively and in real-time. Lightsheet microscopy, Indocyanine green angiography, and histological analyses validated the results generated with this novel method. 71 MHz UHF imaging with the highest array-based ultrasound resolution currently available, enables the visualization of microvessels and their development in-ovo. Contrary to the widespread assumption that gas exchange and nutrient supply of the human cystic tissue on the CAM happens via diffusion, this study demonstrates that vascular perfusion already occurs within a very short period. Therefore, this 3D-in-vivo model in combination with UHF imaging offers promising prerequisites for the visual documentation of angiogenesis, formation of anastomoses, and the high-throughput assessment of effects of possible therapies, potentially facilitating the discovery of personalized therapies in the future.

### Supplementary Information

Supplementary Legends.

Supplementary Video 1.

Supplementary Video 2.

Supplementary Video 3.

Supplementary Video 4.

Supplementary Video 5.

Supplementary Video 6.

Supplementary Video 7.

Supplementary Video 8.

Supplementary Video 9.

Supplementary Video 10.

Supplementary Video 11.

## Data Availability

The datasets used and analysed during the current study are available from the corresponding author on reasonable request.
