# Supplementary material for: Ultra high frequency ultrasound enables real-time visualization of blood supply from chorioallantoic membrane to human autosomal dominant polycystic kidney tissue
Source: Sci Rep. 2024 May 2;14:10063. doi: 10.1038/s41598-024-60783-3 (PMC11066115; doi:10.1038/s41598-024-60783-3)
Supplement: Supplementary file 1 — Supplementary Legends. [file 41598_2024_60783_MOESM1_ESM.docx]

Supplementary file including legends for the supplementary videos

**Supplementary Video 1**

UHF ultrasound of human ADPKD tissue sample on the CAM 12 hours after engraftment (left) and virtual angiography for detecting blood vessels (right). Larger CAM vessels are highlighted in red. Microvessels are highlighted in blue. The millimeter scale (right) shows penetration depth and scale.

**Supplementary Video 2**

UHF ultrasound of human ADPKD tissue sample on the CAM 12 hours after engraftment (left) and virtual angiography for detecting blood vessels (right). Larger CAM vessels are highlighted in red. Microvessels are highlighted in blue. The millimeter scale (right) shows penetration depth and scale.

**Supplementary Video 3**

UHF ultrasound of human ADPKD tissue sample on the CAM 2 days after engraftment (left) and virtual angiography for detecting blood vessels (right). Larger CAM vessels are highlighted in red. Microvessels are highlighted in blue. The millimeter scale (right) shows penetration depth and scale.

**Supplementary Video 4**

UHF ultrasound of human ADPKD tissue sample on the CAM 3 days after engraftment (left) and virtual angiography for detecting blood vessels (right). Larger CAM vessels are highlighted in red. Microvessels are highlighted in blue. The millimeter scale (right) shows penetration depth and scale.

**Supplementary Video 5**

UHF ultrasound of human ADPKD tissue sample on the CAM 4 days after engraftment (left) and virtual angiography for detecting blood vessels (right). Larger CAM vessels are highlighted in red. Microvessels are highlighted in blue. The millimeter scale (right) shows penetration depth and scale.

**Supplementary Video 6**

UHF ultrasound of human ADPKD tissue sample on the CAM 5 days after engraftment (left) and virtual angiography for detecting blood vessels (right). Larger CAM vessels are highlighted in red. Microvessels are highlighted in blue. The millimeter scale (right) shows penetration depth and scale.

**Supplementary Video 7**

UHF ultrasound of human ADPKD tissue sample on the CAM 6 days after engraftment (left) and virtual angiography for detecting blood vessels (right). Larger CAM vessels are highlighted in red. Microvessels are highlighted in blue. The millimeter scale (right) shows penetration depth and scale.

**Supplementary Video 8**

UHF ultrasound of human ADPKD tissue sample on the CAM 7 days after engraftment (left) and virtual angiography for detecting blood vessels (right). Larger CAM vessels are highlighted in red. Microvessels are highlighted in blue. The millimeter scale (right) shows penetration depth and scale.

**Supplementary Video 9**

UHF ultrasound of the CAM and its vessels at a frequency of 71 MHz. The millimeter scale (right) shows penetration depth and scale.

**Supplementary Video 10**

UHF Ultrasound of the CAM and its vessels at a frequency of 46 MHz. The millimeter scale (right) shows penetration depth and scale.

**Supplementary Video 11**

Ultrasound of the CAM and its vessels at a frequency of 22 MHz. The millimeter scale (right) shows penetration depth and scale.
